# Spatial analysis of dengue transmission in an endemic city in Brazil reveals high spatial structuring on local dengue transmission dynamics

**DOI:** 10.1038/s41598-024-59537-y

**Published:** 2024-04-18

**Authors:** André S. Leandro, Wagner A. Chiba de Castro, Michel Varajão Garey, Rafael Maciel-de-Freitas

**Affiliations:** 1 Laboratório de Mosquitos Transmissores de Hematozoários, Instituto Oswaldo Cruz, Fiocruz, Rio de Janeiro Brazil; 2 Centro de Controle de Zoonoses, Secretaria Municipal de Saúde de Foz do Iguaçu, Foz do Iguaçu, Brazil; 3 Universidade Federal da Integração Latino-Americana, Foz do Iguaçu, Brazil; 4 Department of Arbovirology, Bernhard-Nocht Institute for Tropical Medicine, Hamburg, Germany

**Keywords:** Infectious diseases, Parasitology, Pathogens

## Abstract

In the last decades, dengue has become one of the most widespread mosquito-borne arboviruses in the world, with an increasing incidence in tropical and temperate regions. The mosquito *Aedes aegypti* is the dengue primary vector and is more abundant in highly urbanized areas. Traditional vector control methods have showing limited efficacy in sustaining mosquito population at low levels to prevent dengue virus outbreaks. Considering disease transmission is not evenly distributed in the territory, one perspective to enhance vector control efficacy relies on identifying the areas that concentrate arbovirus transmission within an endemic city, i.e., the hotspots. Herein, we used a 13-month timescale during the SARS-Cov-2 pandemic and its forced reduction in human mobility and social isolation to investigate the spatiotemporal association between dengue transmission in children and entomological indexes based on adult *Ae. aegypti* trapping. Dengue cases and the indexes Trap Positive Index (TPI) and Adult Density Index (ADI) varied seasonally, as expected: more than 51% of cases were notified on the first 2 months of the study, and higher infestation was observed in warmer months. The Moran's Eigenvector Maps (MEM) and Generalized Linear Models (GLM) revealed a strong large-scale spatial structuring in the positive dengue cases, with an unexpected negative correlation between dengue transmission and ADI. Overall, the global model and the purely spatial model presented a better fit to data. Our results show high spatial structure and low correlation between entomological and epidemiological data in Foz do Iguaçu dengue transmission dynamics, suggesting the role of human mobility might be overestimated and that other factors not evaluated herein could be playing a significant role in governing dengue transmission.

## Introduction

Dengue virus (DENV) is among the most widespread mosquito-borne arbovirus in the world and its global range has increased since the 1990s^1,2^. DENV is currently present in 128 countries, with almost 4 billion people are estimated to live in areas with risk of infection, with projected estimates showing an increasing trend in illness, fatalities, and geographic range of disease transmission^1–3^. In the last decade, two other arboviruses have dramatically increased their global distribution from restricted regions to a worldwide distribution. Zika (ZIKV) was originally reported in a primate in Uganda in 1947^4^. Large outbreaks were recorded in 2007 in the Pacific Islands^5^, and in the Americas a few years later^6^. The outcomes of ZIKV infection are linked with adverse fetal outcomes such as microcephaly in newborns and Guillain–Barré syndrome, which led the World Health Organization to declare global public health emergency^5^. Chikungunya (CHIKV) was first identified in 1952–1953 during an outbreak in Tanzania^7^. The increased frequency of CHIKV outbreaks in the past 15 years is associated with the spread of the virus to previously non-endemic regions^8,9^. Chikungunya can cause acute symptoms and long-term, debilitating complications such as persistent arthritis, with large disability-adjusted life year estimates (DALY) impacts^10^.

The domesticated species *Aedes aegypti* primarily transmits the three aforementioned arboviruses. This species thrives in urbanized areas since it is adapted to live in close association with human dwellings. Female mosquitoes preferentially blood feed on human hosts^11^, rest inside premises, and lay their eggs mostly in man-made containers located in peridomestic areas^12,13^. Therefore, these bionomic factors make the household the primary location for arbovirus transmission in urban settings^14,15^. Considering the low efficacy of the commercially licensed dengue vaccine and the absence of prophylactic and therapeutic medication for these arboviruses, vector control remains the principal method for DENV, ZIKV, and CHIKV outbreaks mitigation^16,17^.

Traditional vector control tools rely basically on targeting *Ae. aegypti* breeding sites and applying chemical insecticides to maintain mosquito population at low densities to avoid dengue outbreaks^18^. However, container elimination over the long-term in metropolitan areas is unfeasible and the spread of insecticide resistance alleles in *Ae. aegypti* field populations jeopardize the effectiveness of traditional methods relying on insecticide applications in avoiding outbreaks^19,20^. Therefore, there is an urgent need to promote the optimization of vector control actions to boost its effectiveness. One perspective to reframe vector control activities relies on identifying the areas within an endemic city/region that concentrate the local arbovirus transmission to be further prioritized accordingly^21^. The temporal and spatial scale at which arboviral diseases cluster is informative about the underlying transmission dynamics and provide the opportunity to prioritize high-transmission areas to foster adequate intervention and interrupt transmission^21–24^. Vector control is traditionally reactive, responding to clinically apparent disease manifestation in the case’s primary residence^25^. However, determining the precise location of arbovirus transmission is challenging mostly due to the relevance of human movement within DENV, ZIKV, and CHIKV transmission as outlined by both observational studies and mathematical models, but also the role of asymptomatic hosts in dengue transmission^14,26–28^. Since human exposure to infectious mosquitoes and virus transmission by viremic individuals can be spatially disassociated^27^, endemic cities with high human mobility have additional hurdles in defining disease transmission clusters. Therefore, the appropriate identification of subareas within endemic cities that asymmetrically concentrates arbovirus transmission consists in additional challenge for managing dengue outbreaks. Furthermore, if those disease transmission hotspots are timely identified, local public health managers could target those regions under higher risk by intensifying vector control activities on them. For example, ultra-low volume adulticides, targeting breeding sites and enhance community mobilization and sensitivity to good practices could promote an overall more effective response in comparison with a citywide approach that does not consider local heterogeneity^23,29–31^.

The surge of SARS-CoV-2 have also impacted surveillance and response for other infectious diseases. In the case of arboviruses, disease notification decreased, and surveillance was hindered by the avoidance of health agents in entering people’s house. On the other hand, among the consequences of the SARS-CoV-2 pandemic is the reduction of human mobility in response to containment measures, e.g., social isolation. Schools and children had to adapt to online remote classes between 2020–2021, i.e., children’s mobility was insignificant during this period. Therefore, we investigated the spatiotemporal association between dengue transmission in children and entomological indexes within this specific timeframe in the endemic city of Foz do Iguaçu, Brazil, after intense adult *Ae. aegypti* trapping across the study area.

## Results

### Human demography and epidemiological data

**Figure 1:**
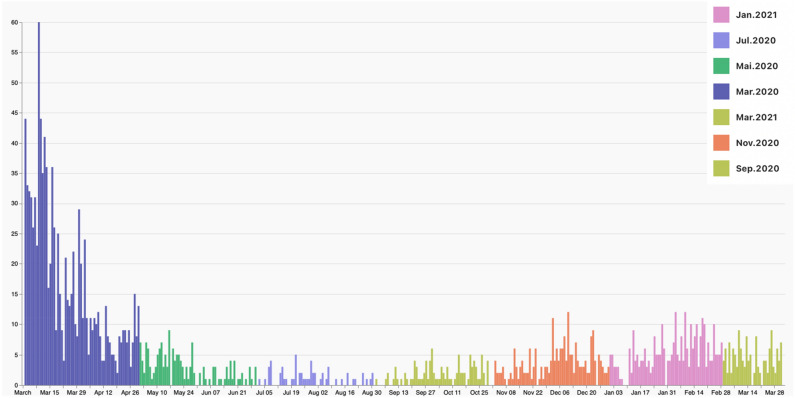
Daily number of dengue cases reported in children with less than 10 years in the city of Foz do Iguaçu between March 2020 to March 2021. This period comprises the SARS-CoV-2 pandemic when children have limited mobility due to social restrictions. Each color represents 2 months of epidemiological data associated with the entomological surveys, except for March 2021, when mobility limitations were relaxed.

**Figure 2:**
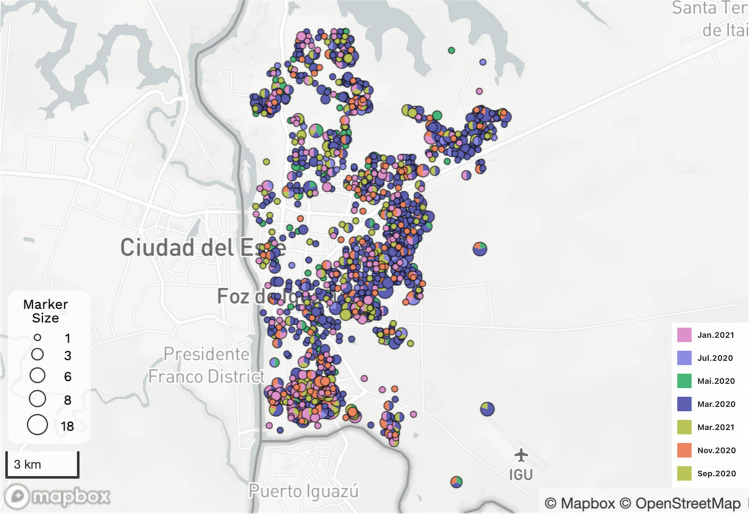
Map of Foz do Iguaçu showing the spatial distribution of dengue cases. Colors in the pie chart are associated with the bimonthly larval surveys, using the same colors as in Fig. 1.

The total human population of Foz do Iguaçu is 258.248 people, with an average density of 414.6 inhabitants per km^2^, considering rural and preserved uninhabited regions. The 73 urban areas enrolled in this investigation had human densities varying between 253 and 9016 hab/km^2^. A total number of 1992 children with less than 10 years enrolled in this study. The spatiotemporal distribution of dengue cases in the selected cohort is available online on the microreact platform (https://microreact.org/project/8DhMNEmMEbFUsvK9xHS349-foz). Dengue incidence had a strong seasonal pattern in Foz do Iguaçu. From the 1992 dengue cases reported in the period, a higher incidence was observed in March 2020, which accounted for 51.15% of the total cases (Fig. 1). Overall, a few spatial clusters of dengue transmission were observed in Foz do Iguaçu, especially on the South and East side of the city (Fig. 2).

### Entomologic data

A total of 3476 adult mosquito traps (Adultrap) is distributed along the city, in all the 73 urban areas of Foz do Iguaçu. Each Adultrap is inspected every 2 months by local health agents during their routine surveillance, i.e., during the period of this study, each trap received seven inspections. Two adult-based indexes performed better in forecasting dengue outbreaks: the trap positive index (TPI) and the adult density index (ADI). TPI refers to the number of positive traps among the total number of traps inspected multiplied by 100, whereas ADI is the total number of *Ae. aegypti* mosquitoes captured divided by the total number of inspected traps multiplied by 100. Both TPI and ADI were calculated for each survey, i.e., every 2 months during the study period.

**Figure 3:**
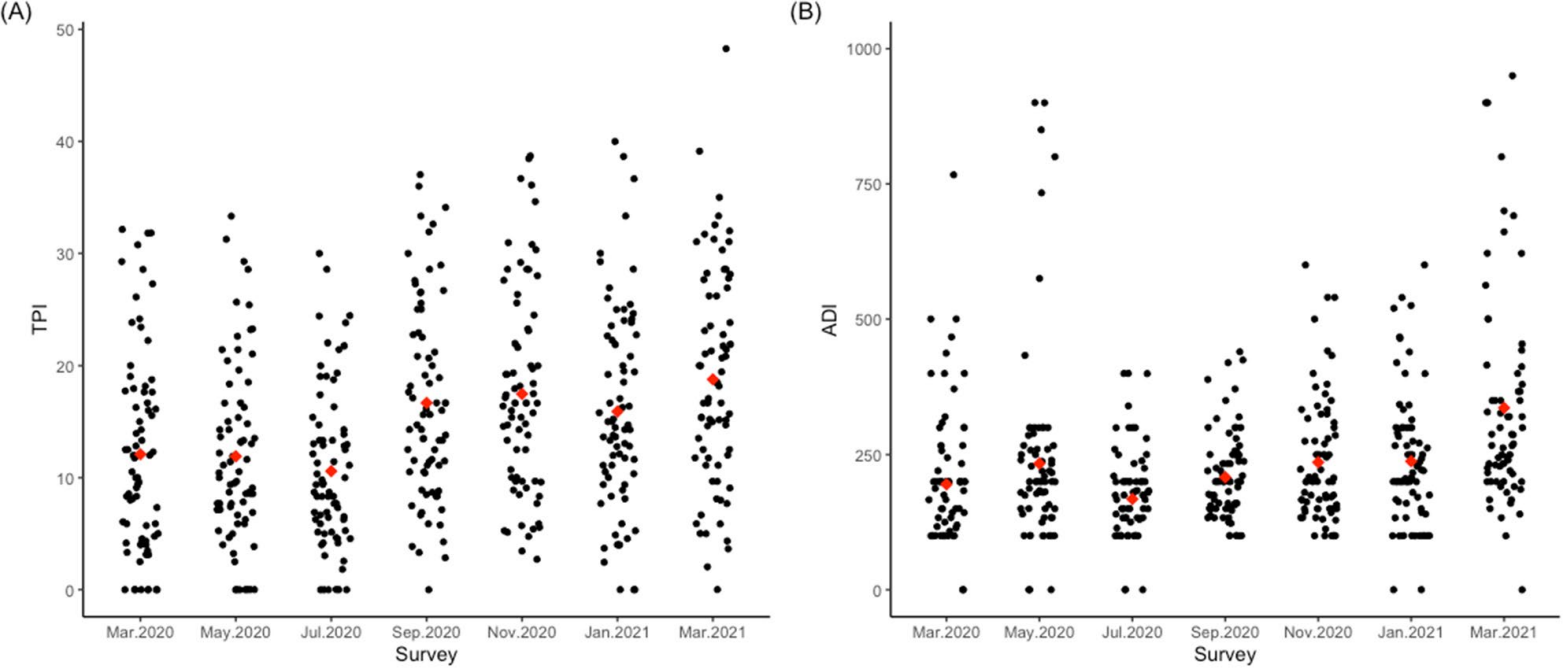
Entomological indexes based on adult *Aedes aegypti* sampling in the city of Foz do Iguaçu between March 2020 to March 2021. (**A**) Trap Positive Index, TPI; (**B**) Adult Density Index, ADI. Each dot represents the index value in one of the 73 study areas, whereas the red dot represents the mean value of each survey.

An average of 69.45 *Ae. aegypti* mosquitoes were captured in each of the 73 areas across the study period. The number of *Ae. aegypti* mosquitoes captured per area ranged from 13 to 198 individuals. Considering 438 observations (six surveys in 73 urban areas), in only 21 of them (4.79%) no *Ae. aegypti* mosquitoes were captured in the traps. The entomological indexes reflected the expected seasonality of mosquito infestation, with the lowest values recorded in the coolest month (July/2020), followed by an increase in the subsequent months (Fig. 3). The highest value for ADI was observed in January/2021; meanwhile TPI present higher values from September/2020 to January/2021 (Fig. 3). During this period, 10 *Ae. aegypti* females were found naturally infected by DENV, whereas 03 for CHIKV, out of a total of 202 samples tested.

### Spatial correlation between entomological and epidemiological data

**Figure 4:**
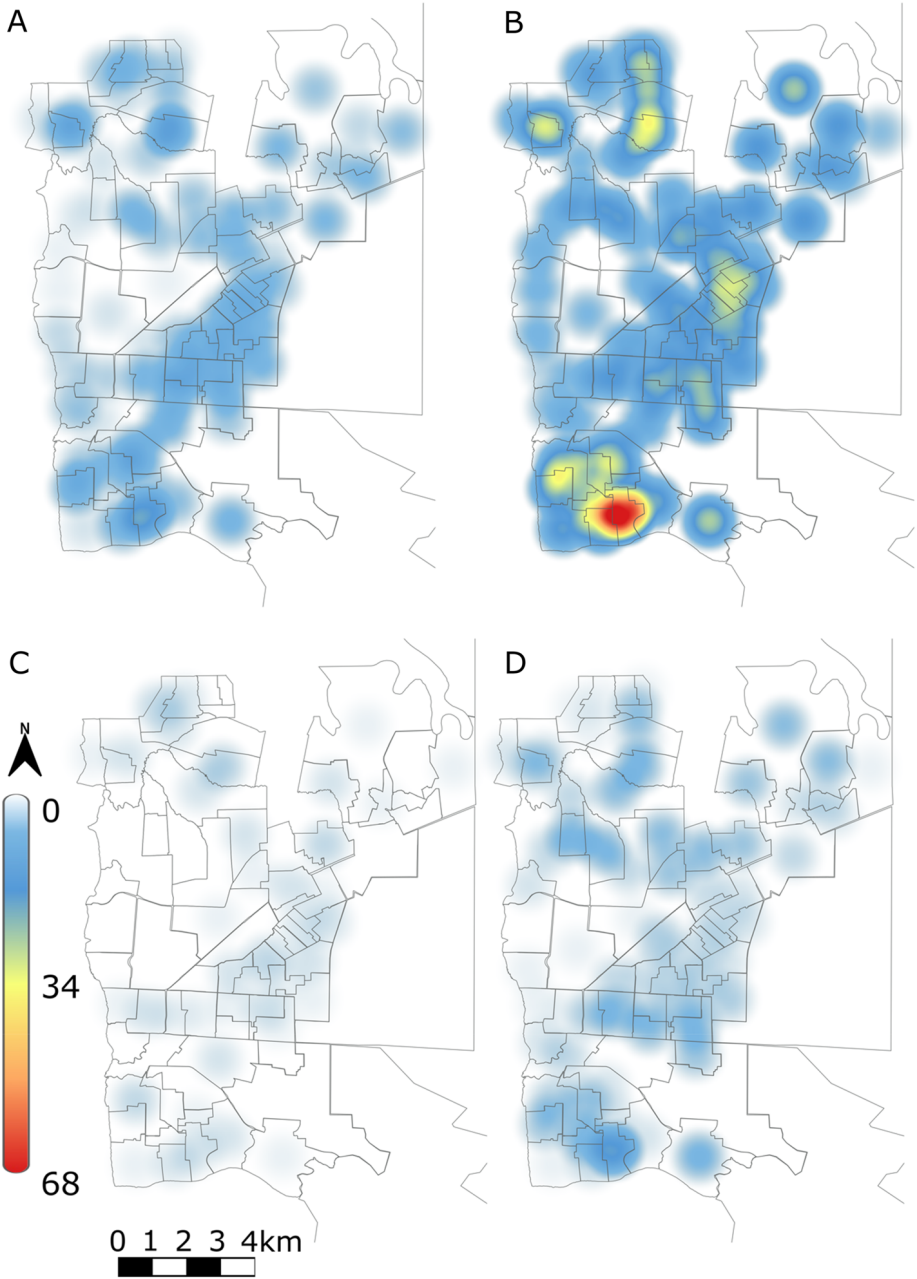
Number of dengue cases reported in children with less than 10 years in the city of Foz do Iguaçu between March 2020 to March 2021. (**A**) Notification; (**B**) Notification_4wk; (**C**) Notification + Confirmation; (**D**) Notification + Confirmation_4wk. Figure was constructed using a Kernel smoothing with quartic function and width of 1000 m. i.e., the influence of a point decreases as the distance from the point increases.

Considering the accumulated dengue data during 13 months per area of the city of Foz do Iguaçu, we obtained that the global model was the best for the total number of cases in children up to 10 years of age (Table 1). Within this model, the variable ADI was negatively correlated with the number of dengue cases, and the spatial vector MEM 2 was also significant, highlighting a large-scale spatial structuring in the positive dengue cases (Fig. 4; Table 2).

**Table 1:** The four models were used to examine the relationship among the accumulative number of positive cases for dengue in children up to 10 years old and spatial and local variables during one epidemiological year in Foz do Iguaçu, Paraná, southern Brazil. The global model encompasses both local and spatial variables. The special model includes only MEMs as predictors. The environmental model incorporates only local variables. The model with intercept accounts for the variation in the response variable when all predictors are equal to zero (i.e., random effects model). ∆AICc = difference in corrected Akaike’s Information Criteria, df = degrees of freedom, Weight = weights of corrected Akaike’s Information Criteria.

	∆AICc	df	Weight
Notification			
Global model	0.0	8	0.882
Spatial	4.1	4	0.116
Environmental	11.6	6	0.002
Intercept	–	2	< 0.001
Notification_4wk			
Global model	0.0	12	0.86
Spatial	3.7	8	0.14
Environmental	48.8	6	< 0.001
Intercept	–	2	< 0.001
Notification + confirmation			
Spatial	0.0	4	0.52
Global model	1.4	8	0.25
Environmental	1.6	6	0.23
Intercept	–	2	< 0.001
Notification + Confirmation_4wk			
Spatial	0.0	4	0.844
Global model	3.4	8	0.152
Environmental	11.1	6	0.003
Intercept	–	2	< 0.001

**Table 2:** P-value of the predictor variables included in the best generalized linear model (see Table Y) obtained through the ANOVA. Pop. Dens. = Population density; Mosquitoes = total of individuals captured in traps; TPFM = Percentage of traps that captured mosquitoes; ADI = the ratio of the number of mosquitoes captured divided by the number of traps that captured mosquitoes; Spatial vectors = Moran Eigenvector Maps considered in each model. Significant values are in bold.

	Pop. dens.	Mosquitoes	TPFM	ADI	Spatial vectors
Notification	0.174	0.841	0.758	0.049	MEM 1 = 0.167 **MEM 2 = 0.0005**
Notification_4wk	0.055	0.003	0.558	0.905	**MEM 4 = < 0.0001** **MEM 3 = 0.0001** **MEM 6 = < 0.0001** MEM 10 = 0.068 **MEM 12 = 0.0004** **MEM 15 = 0.0123**
Notification + Confirmation	0.546	0.494	0.273	0.693	MEM 1 = 0.669 MEM 2 = 0.321
Notification + Confirmation_4wk	–	–	–	–	MEM 1 = 0.384 **MEM 2 = 0.0009**

Concerning the ‘Notification’ plus the ‘Notification_4wk’, we found that the global model was the best model to explain the variation in the number of cases (Table 1). We found that the greater the number of mosquitoes captured, the lower the number of cases recorded. The spatial vectors (MEM 3, 4, 6, 12, and 15) were also significant, stressing spatial structuring at broad and intermediate spatial scales (Table 2; Fig. 4). When analyzing the ‘Notification + Confirmation’, we found that the global, the purely spatial, and purely environmental models were equally parsimonious (Table 1). However, no predictor variable was related to variation in dengue cases (Table 2). Considering ‘Notification + Confirmation’ and the ‘Notification + Confirmation_4wk’, we found that the best model contained only the spatial vectors (Table 1). The MEM 2 vector was significant, highlighting a spatial structuring of dengue cases at broad spatial scales (Table 2).

We found a strong spatial pattern in the distribution of cases when we evaluated the number of dengue cases per each of the routine surveillance periods. In general, we only observed a spatial influence in the first survey (March 2020), where the four response variables were explained solely by spatial factors, highlighting a spatial structure in dengue cases at broad to intermediate scales (Table S1). The spatial effect was also observed in the models developed separately for each subsequent routine surveys. However, ‘Notification + Confirmation’ during the survey on January 2021 was explained both by spatial factors and by ADI and the higher the ADI, the higher the number of cases (Table S1). In the survey of March 2021, the ‘Notification + Confirmation’ was explained by both spatial factors and the population density of each area, so the higher the population density, the higher the number of cases. During July 2020, however, no predictor variable was associated with variation in the response variables.

## Discussion

Over recent decades, mosquito-borne viruses have emerged or reemerged worldwide, posing a threat to global health. Efficient surveillance systems able to constantly monitor vector populations and the first cases of targeted mosquito-borne disease are of the utmost relevance. Ideally, surveillance systems should be strengthened in accordance with the One Health framework so that the different components involved in vector-borne diseases epidemiology can be suitably addressed^32,33^. Regarding dengue transmission at the local level in the city of Foz do Iguaçu, we have been able to develop entomological indicators based on adult sampling in mosquito traps that are able to predict the occurrence of dengue outbreaks in the next 4 weeks, increasing local preparedness for upcoming threats^34^. However, we face limited advances in determining spatial correlations between mosquito indexes based on adult sampling and epidemiological data^35^. Herein, we reported the use of Moran Eigenvector Maps to investigate whether a spatiotemporal association between entomological and epidemiological data exists in analyzing dengue incidence in a specific cohort: children with < 10 years old during the social isolation promoted by the SARS-CoV-2 pandemic, when children had online remote classes and insignificant mobility between 2020–2021. Our results show high spatial structure and low correlation between entomological and epidemiological data in local dengue transmission dynamics. In our approach, we statistically tested the spatial effect on dengue cases. By doing so, we were able to understand that at a time of low displacement rates, cases are concentrated in some areas, unrelated to environmental variables. Likewise, the more cases in one area, the greater the chance of more cases in neighboring areas.

The epidemiological surveillance of dengue endemic countries is most often based on reacting to local householders seeking assistance in the Municipality health network. A subset of 33.2% of children involved in our study were further screened for molecular confirmation of dengue infection. Foz do Iguaçu screen around 40% of people seeking for assistance in the Municipality Health network, which is considered a number far beyond the national average^36^. In the absence of confirmation, one cluster was determined in the Southern region of the city (Fig. 4B). This region has been recording the highest incidence of dengue transmission in previous years and is also the place for high *Ae. aegypti* populations^34,35^. It has some attributes that could favor *Ae. aegypti* installment and its maintenance, such as higher human density, moderate vegetation coverage, non-regular water distribution, and high availability of mosquito breeding sites^13,23,37,38^. Most importantly, our results show that this is a region of higher vulnerability for dengue transmission and must receive continuous support from the local public health initiatives and vector control interventions.

An effective Early Warning System (EWS) for arboviruses should be able to correctly provide spatiotemporal predictions allowing mapping of high transmission areas at small spatial levels^39,40^. A recent review summarized evidence of EWS for mosquito-borne diseases in different terms and concluded that spatial prediction remains a limitation, with no tool currently able to map high transmission areas at a small spatial level^41^. In this review, a total of 24 EWS developed for DENV were scrutinized, and 20 of them demonstrated high temporal prediction ability, whereas only three showed high spatial prediction behavior^42–44^. A forecasting algorithm using spatiotemporal data was able to derive dynamic risk maps for dengue transmission at a neighborhood level resolution (1 km^2^) in Singapore^42^. The China Infectious Disease Automated-alert and Response System (CIDARS) was implemented nationwide in 2008 and used time series moving percentile of historical data to identify dengue fever outbreaks reported from 2009 to 2012 with a sensitivity of 100%, a specificity of 99.8% and a median time to detection of 3 days^43^. Retrospective and prospective analyses of time series combined with spatial statistics comprising entomological and climatic indicators were able to provide alerts of changes in vector populations at a 20 × 20 km resolution in Cuba^44^. Overall, despite providing spatial analysis is crucial for further directing effective vector control interventions, available EWS often are not able to provide spatial prediction to determine disease transmission hotspots^41^.

The role of human mobility in the DENV transmission dynamic has been outlined by both field observational studies and mathematical modeling^28,45^. Using data gathered on a case–control study design and 54 contact-site cluster investigations over two dengue transmission seasons in Peru, authors showed that infection risk and transmission rates are substantially elevated among households visited, highlighting the role of human movement on local dengue spatiotemporal dynamics^26^. By performing a contact tracing approach in the city of Cairns, Australia, the local DENV transmission network during an epidemic was investigated, and it revealed that human mobility was a major mechanism responsible for long-distance virus propagation. On the other hand, the probability of detecting contact locations was negligible within close proximity of the home residence, set as below 200 m^25^. Overall, results gathered in Cairns agree with published results showing the relevance of human mobility and highly focal DENV transmission from urban areas in Peru and Vietnam^25,26,46^. Using a cohort of children < 10 years during the mobility restriction imposed by the SARS-CoV-2 pandemic does not provide insightful spatial correlations between entomological indexes and dengue notifications in the city of Foz do Iguaçu. We acknowledge that our approach has several limitations, but nonetheless, it provided valuable information for understanding the local spatial dimension of dengue transmission in a specific timeframe. One hypothesis to explain an apparent low or absent effect of human mobility in dengue transmission might be a natural consequence of the criteria adopted for cohort selection. By selecting only < 10 years-old children, we did not included adults in our cohort, i.e., we have no information whether the parents of the 1992 children participating in the study had any mobility restriction during the study period. Thus, it is possible that, for instance, high mobility from their parents could have reduced the isolation of the children that belong to our cohort.

One of the most critical activities in arbovirus epidemiology involves determining the hotspots of disease transmission in urban metropolitan areas. Dengue transmission is usually heterogeneous over urban settlements and local public health managers would benefit if there is a timely tool to identify specific regions within the city limits where dengue cases pop out more intensely. For example, if the hotspot of dengue transmission in the next mosquito season is well known, as the intensification of vector control interventions in the areas with higher risk can be adopted^40,47^. Therefore, if effective, it is reasonable to expect the intensification of vector control activities in the high-risk areas would produce a positive impact in dengue transmission over the city^21^.

Regarding dengue transmission in endemic settings, attention has been placed on mapping the most productive breeding sites, key premises, and houses more likely to harbor high populations of *Ae. aegypti*, entomo-virological surveillance, massive trapping, satellite images, and modeling^23,36,48–56^. Using data from Foz do Iguaçu, Brazil, we are able to predict an increase in dengue cases with 4 weeks in advance but face no success in providing spatial prediction to local health managers^34,35^. One remarkable observation about our dataset is the overall lack of correlation between entomological predictors and dengue notification in our study cohort. A previous multicentric study comparing several traps in Brazil with traditional larval surveys revealed that any of mosquito traps performed better than the larval surveys in providing alert signals for dengue outbreaks^52^. Although the Adultrap was capable of detecting large mosquito variations throughout two consecutive years in a consistent way, it is a trap with lower efficacy in the field^34,57,58^. Additionally, several factors we did not control can affect trap efficiency, such as the abundance of container types nearby and human needs for water storage^13,37,59^. Therefore, any extrapolation of our results must be taken carefully since it might be specific to Foz do Iguaçu and its health unit distribution, mosquito infestation patterns, and trap used.

The data gathered in Foz do Iguaçu shows that dengue transmission in this local context presents a high spatial structure and low correlation with entomological indicators. The lack of spatial correlation between dengue cases and entomological indicators for the < 10 years-old children reinforces that effective results against arboviruses will be solely achieved by integrative vector management approaches^60^. Foz do Iguaçu city has a citywide surveillance system operating since 2017 that is based on 3500 adult mosquito traps and screening for DENV, ZIKV, and CHIKV on both mosquitoes sampled alive and symptomatic humans nearby traps with results 36h after sampling^33–35^. Therefore, local public health managers could promote more effective dengue control by improving surveillance of the first human cases detected in the early beginning of the dengue season^61–63^. In a highly spatially structured dengue transmission context, as observed in Foz do Iguaçu, the exponential increase of dengue cases seen in an outbreak could be halted if the intensification of vector interventions on the surroundings of the first reported cases is effective^30^.

## Materials and methods

### Study site

The study was conducted in the city of Foz do Iguaçu (25°30′58″ S, 54°35′07″ W), Brazil. It is among the most visited cities in Brazil and is located on the Triple Border with Paraguay and Argentina. It is reasonably well isolated since on its west side there is the Paraná River, on the North there is the Itaipu hydroelectric power plant and the Iguazu National Park on the South. Nevertheless, intense movement is observed especially between Brazil and Paraguay, mostly for trading and smuggling. Foz do Iguaçu has ≈ 260,000 inhabitants and is divided into 73 urban areas of ≈ 1500 premises each, plus three rural areas were not included in this study. The human population is presented in an average of 3522 ± 1096 (mean ± SD) people per the 73 monitored study areas. The climate in Foz do Iguaçu is classified as humid tropical, according to the Köppen–Geiger system, and is characterized by hot and humid summers (mean temperature > 27 °C) and cold to mild winters (mean temperature of 17 °C) and an average annual rainfall of 1850 mm^64^. The city is endemic for dengue transmission since the 1990s and experiences dengue outbreaks every 4–5 years^33^.

### Adult mosquito collection

Since January 2017, 3476 Adultraps have been installed in the city on a ratio of one trap for every 25 premises. This trap is designed to preferentially capture gravid *Ae. aegypti* female mosquitoes during oviposition as it uses water as the principal attractant. These traps have an opening on the top where females enter, then are trapped in an interior chamber^57^. Water remains confined in a compartment at the bottom of the trap that the mosquitoes cannot access, thus deterring egg-laying. Local health agents visit all Adultraps every 2 months during their routine surveillance activity, which consists of larval surveys and trap inspections in the houses where access is granted. Trap inspection and larval surveys occur on the first 2 weeks of every odd month, i.e., there is an average period between 6–7 weeks between surveys. The analyses reported herein are limited to the period between March 2020 to March 2021 to cover a period in which children’s mobility was almost inexistent due to the need for social isolation and online remote classes. During these 13 months, seven surveys were conducted in each of the 73 areas (March, May, July, September, and November 2020; January and March 2021), totalizing 438 observations, resulting in more than 20,000 trap inspections.

### Entomologic indices

Analyzing data gathered in Foz do Iguaçu from 2017–2020; we showed that entomological indices based on adult *Ae. aegypti* sampling has a superior capacity to predict the occurrence of dengue outbreaks 4 weeks in advance than traditional indexes such as House Index (HI) and Breteau (BI)^34^. Likewise, among the adult-based indexes, two of them performed better in forecasting dengue outbreaks: the trap positive index (TPI) and the adult density index (ADI). TPI refers to the number of positive traps among the total number of traps inspected multiplied by 100, whereas ADI is the total number of *Ae. aegypti* mosquitoes captured divided by the total number of inspected traps multiplied by 100 had better forecasting performance and were thus selected for further analysis. We calculated both TPI and ADI every 2 months during the study period, totalizing seven observations.

### Entomo-virological surveillance

This activity consisted of screening those mosquitoes identified as *Ae. aegypti* that were trapped alive in the Adultraps^34,35^. Mosquitoes were collected in the field, identified using adequate taxonomic keys, and counted. Dead individuals were discarded, whereas those still alive were screened by RT-qPCR for ZIKV, CHIKV, and DENV, including serotype identification if DENV-infected. Viral RNA extraction from field-caught *Ae. aegypti* used the MagMAX Viral/Pathogen Nucleic Acid Ultra Isolation KIT, according to the manufacturer’s instructions. Mosquitoes were individually added to tubed with electromagnetic mixing beads macerated using TissueLyser II. For arboviral genome amplification, we used the ZDC Biomol Kit, which enables the identification of ZIKV, CHIKV, and differentiation of DENV serotypes with an internal control (IC) of the reaction that uses probes specific to each molecular target. We used a 96-well QuantStudio 7 Flex Real-Time PCR System for PCR and analyzed results using QuantStudioDesign and Analysis Software versions 1.3.1 and 1.5.1. Samples were considered positive when the amplification plot curve exceeded the specific threshold for each target < 35 cycle threshold. Results from the entomo-virological surveillance are available in less than 36 h after mosquitoes are collected in field traps^33^. An average natural infection rate of 13.1% was observed in Foz do Iguaçu between 2017–2020, with 75.9% of the positive pools diagnosed as DENV-infected^34^.

### Epidemiological data

The health system in Foz do Iguaçu is homogeneously distributed within the city limits and is currently composed of 30 basic health units, including two emergency care units and four hospitals (three private and one public). The Ministry of Health lists dengue as a disease of compulsory notification that can be registered in any local health facility. Epidemiological data is recorded in the available at the Information System for Notifiable Diseases (SINAN) and is available to the public health team of every Brazilian city. The raw data from Foz do Iguaçu was filtered according to the period (03.2020–03.2021), birth date selecting only those with < 10 years, and DENV diagnostic based on either clinical-epidemiological or molecular tests (RT-qPCR). Epidemiologic surveillance for arboviruses in Brazil is reactive and is carried out passively after symptomatic persons seek care in the city health system. A suspected dengue case was reported whenever any person residing in Foz do Iguaçu received a clinical diagnosis of ≥ 1 compatible dengue symptom, including fever, headache, myalgia, arthralgia, rash, nausea, retro-orbital pain, petechiae, or malaise, in the previous 14 days, following the National Guidelines from Brazilian Ministry of Health^65^. Among suspected dengue cases, a subset of 33.2% were further screened for dengue through laboratory diagnosis. The epidemiological data used in this manuscript was the number of dengue reported cases in children up to 10 years-old in the four subsequent weeks immediately after the entomological survey. The Supplementary Table 2 (Table S2) summarizes how entomological and epidemiological data were managed during the study period.

### Patient inclusion criteria

In order to reduce or eliminate the effect of urban mobility and seek to associate arboviruses notification with the presence of the vector *Ae. aegypti*, we used data only from children up to 10 years of age confirmed positive for dengue by laboratory or clinical-epidemiological criteria from March 2020 to March 2021. During this period, all schools remained closed, and children stayed at their homes with online classes due to a national recommendation for social isolation established due to SARS-CoV-2 pandemic. We considered as positive cases those symptomatic individuals whose blood was screened for DENV through RT-qPCR in the Central Laboratory of the State of Paraná (LACEN Paraná) and those who had a spatiotemporal relationship with another case of confirmed dengue infection, e.g., another member of the family within seven days of the laboratory-confirmed case.

### Spatial data

We used Moran Eigenvector Maps (MEM) analysis to obtain the spatial variables. MEM is a multiscale ordination method, generating orthogonal eigenvectors employed for depicting spatial relationships among the 73 urban areas within in Foz do Iguaçu, providing a set of spatial predictors^66^. The application of MEM enables the statistical computation of spatial effects on dengue cases, as the axes generated from the ordination capture independent spatial variations that represent the distance and spatial configuration of 73 urban areas. The MEM analysis entails the Principal Coordinates Analysis of a truncated matrix generated from the geographical coordinates of sites, employing Euclidean distance, a connectivity matrix, and a weighted spatial matrix. The first axes of an ordination, in this case MEM, concentrate the majority of spatial variation, describing broad spatial structures that encompass spatial variation across the entire study area^66^. Conversely, the latter eigenvectors depict finer spatial structures that capture variation at the local scale, i.e., among areas that are geographically closer^66^. In this manner, within the models, we must possess spatial eigenvectors that represent distinct spatial scales, ranging from the finest to the broadest. MEMs were obtained following the protocol proposed by Bauman et al.^67^, which estimates and selects a set of MEMs that describes the best subset of spatial variables in representing spatial patterns in the positive dengue cases in each area. This spatial optimization protocol is achieved through direct selection with double-stopping criteria^68^, aiming to select the optimal subset of MEM variables based on the highest adjusted R^2^ value^67^. Because this procedure involves multiple tests, we used Sidak’s correction to adjust the *P* values^67^. By applying this protocol, we obtained the MEM variables of the best model as a result, which were used as spatial predictor variables in subsequent analyses. When no model was selected, we retained the first two axes of the MEM (i.e., the axes that capture the largest spatial variations in the data) so that spatial variables were included in all models.

### Data analysis

In order to verify whether the variation in the number of dengue cases among the 73 areas is associated with local factors or spatial variation, we performed Generalized Linear Models (GLM) analysis. The analyses were performed in two different temporal clippings The first considered the number of positive cases accumulated over 13 months (March 2020 to March 2021). The second temporal clipping consisted of partitioning these 13 months into seven almost equal time intervals (March, May, July, September, and November 2020; January and March 2021). These intervals correspond to routine surveillance activities conducted by public health agents in inspecting the Adultraps for mosquitoes. The temporal partition aimed to investigate whether, at certain times of the year, the mosquito abundance caught could be related to dengue cases. Thus, in total, the analyses were conducted considering eight-time clipping. Within each time clipping, we evaluated the effects of local and spatial variables (predictor variables) on four different response variables separately for each time interval. The response variables considered in the present study were:: (i) total notifications of dengue positive, named hereafter ‘Notification’; (ii) cumulative number of positive dengue cases reported in the next 4 weeks, named ‘Notification_4wk’; (iii) notifications of dengue positive with the confirmation of the laboratory and clinical examination, named ‘Notification + Confirmation’; and (iv) cumulative notifications of positive dengue in the next 4 weeks with the confirmation of the laboratory and clinical examination, named ‘Notification + Confirmation_4wk’. Based on our previous findings showing a high correlation between entomological indexes and dengue cases in the following 4 weeks^34^, we used as response variables dengue cases recorded in the same week of traps inspection (i and iii described above) but also used the cumulative notifications in the subsequent 4 weeks. In the GLM, we considered the spatial variable (MEMs) and four local variables from each area to predict the variation in the aforementioned response variables, namely: (i) human population density in each area; (ii) total number of mosquitoes captured in all traps within the same area; (iii) percentage of traps within the same area that captured *Ae. aegypti* mosquitoes (TPI); and (iv) the ratio of the number of mosquitoes captured divided by the number of traps that captured mosquitoes (ADI). As previously reported, the spatial predictor variables inserted in the models were obtained through Moran’s Eigenvector Maps (MEM). For each response variable considered, four independent models were constructed. In short, we evaluated the effects of local and spatial variables on four different response variables, totaling four GLM for each response variable. However, this procedure was repeated considering the data accumulated over 13 months and subsequently for each routine surveillance, totaling 32 GLMs, which are explained below.

In GLMs analyses, we first built a global model for each predictor variable, that is, a model that includes all local (i.e., human population density, number of mosquitoes captured, TPI and ADI) and spatial predictor variables (i.e., selected MEMs), using a Poisson distribution to check for the presence of overdispersion. If the global model showed overdispersion, we used a Negative Binomial distribution with a log-type link function. To verify that the overdispersion problem was solved using models with a Negative Binomial distribution, we used residual analysis using the KS test, dispersion, and outlier test, and in all the best models obtained, the cases of overdispersion were solved. Subsequently, we built three other models: (i) a model containing only the local variables of each area (i.e., human population density, number of mosquitoes captured, TPI and ADI); (ii) a model containing only the spatial predictors (i.e., selected MEMs); and (iii) a model containing only the intercept (i.e., the predicted value of the response variable when all the predictor variables are zero). Thus, four models were constructed for each response variable, which were comparatively evaluated from the relative support of each model from Akaike's Information Criteria (ΔAICc) and Akaike's weights^69,70^. We applied criteria based on likelihood-based inference^33^ for selecting the best model, using the evidence ratios, i.e., the relative likelihood of model i versus model j^70^. Akaike weights were used to assess the uncertainty of model selection, quantifying the probability that the model is the best among all models built for that response variable^69,70^. For each set of models created for a given response variable, we selected the best model(s) for interpretation of its parameters if: (i) had ΔAICc less than 2.0, and (ii) were included in the set of best-supported models with combined Akaike weights greater than 0.70 (confidence greater than 70%)^69,70^.

### Ethical issues

All activities regarding vector-borne diseases surveillance and control are performed by the health agents of the Centro de Controle de Zoonoses da Secretaria Municipal de Saúde de Foz do Iguaçu (CCZ-Foz do Iguaçu). Among the actions carried out by health agents in their daily routine are larval surveys, an inspection of Adultraps, and access SINAN website for gathering epidemiological data. Due to this surveillance routine, many residents in the study area are used to having mosquito traps installed in their houses, and, indeed, there were just a few refusals by residents to have traps installed. Written consent was not required under the ethical committee requirements. As the effort to collect mosquitoes goes along with city surveillance, these tasks are typically done with oral consent only, and our collective effort also had oral consent. The entomology field team registered individual consents in regular work files.

### Supplementary Information

Supplementary Tables.

## Data Availability

The datasets used and/or analysed during the current study available from the corresponding author on reasonable request.
