# Supplementary material for: Spatial analysis of dengue transmission in an endemic city in Brazil reveals high spatial structuring on local dengue transmission dynamics
Source: Sci Rep. 2024 Apr 18;14:8930. doi: 10.1038/s41598-024-59537-y (PMC11026424; doi:10.1038/s41598-024-59537-y)
Supplement: Supplementary file 1 — Supplementary Tables. [file 41598_2024_59537_MOESM1_ESM.pdf]

Supplementary Table 1: The four models used to examine the relationship among the accumulative number of positive cases for dengue in children up to ten years old, spatial and local variables during seven bimonthly monitoring between March 2020 to March 2021 in Foz do Iguaçu, Paraná, southern Brazil. ΔAICc = difference in corrected Akaike's Information Criteria; df = degrees of freedom; Weight = weights of corrected Akaike's Information Criteria.

		ΔAIC	df	Weight
Survey 1	Notification			
March 2020		0	16	0.532
	Spatial	0.3	12	0.470
	Environmental	12.3	6	0.001
	Intercept	-	2	<0.001
	Notification_5wk			
	Global model	0	16	0.810
	Spatial	2.9	12	0.190
	Environmental	23.4	6	<0.001
	Intercept	-	2	<0.001
	Notification+Confirmation			
	Global model	0	15	0.630
	Spatial	1.1	11	0.370
	Environmental	22.6	5	<0.001
	Intercept	-	2	<0.001
	Notification+Confirmation_5wk			
	Global model	0	16	0.718
	Spatial	1.9	12	0.281
	Environmental	11.9	6	0.002
	Intercept	-	2	<0.001
Survey2	Notification			
May 2020	Global model	0.0	16	0.87
	Spatial	3.8	12	0.13
	Environmental	17.7	5	<0.001
	Intercept	-	2	<0.001
	Notification_5wk			
	Global model	0.0	17	0.956
	Spatial	6.3	13	0.041
	Environmental	11.6	6	0.0029
	Intercept	-	2	<0.001
	Notification+Confirmation			
	Global model	0.0	16	0.938
	Spatial	5.4	12	0.062
	Environmental	16.5	5	<0.001
	Intercept	-	2	<0.001

	Notification+Confirmation_5wk			
	Global model	0.0	16	0.916
	Spatial	5.6	12	0.057
	Environmental	7.0	5	0.028
	Intercept	-	2	<0.001
Survey3	Notification			
July 2020	Global model	0.0	18	0.639
	Spatial	1.2	14	0.350
	Environmental	8.1	5	0.011
	Intercept	-	2	<0.001
	Notification_5wk			
	Global model	0.0	18	0.75
	Spatial	2.2	14	0.25
	Environmental	13.9	5	<0.001
	Intercept	-	2	<0.001
	Notification+Confirmation			
	Spatial	0.0	14	0.53
	Global model	0.3	18	0.47
	Environmental	18.9	5	<0.001
	Intercept	-	2	<0.001
	Notification+Confirmation_5wk			
	Global model	0.0	18	0.686
	Spatial	1.9	14	0.261
	Environmental	5.1	5	0.053
	Intercept	-	2	<0.001
Survey4	Notification			
September 2020	Global model	0.0	15	0.535
	Spatial	0.4	11	0.449
	Environmental	7.0	5	0.016
	Intercept	-	2	<0.001
	Notification_5wk			
	Global model	0.0	16	0.770
	Spatial	2.5	12	0.23
	Environmental	35.6	6	<0.001
	Intercept	-	2	<0.001
	Notification+Confirmation			
	Global model	0.0	15	0.860
	Spatial	3.7	11	0.137
	Environmental	11.3	5	0.003
	Intercept	-	2	<0.001
	Notification+Confirmation_5wk			

	Global model	0.0	16	0.690
	Spatial	1.6	12	0.310
	Environmental	21.1	6	<0.001
	Intercept	-	2	<0.001
Survey5	Notification			
November 2020	Global model	0.0	15	0.790
	Spatial	2.6	11	0.210
	Environmental	13.4	5	<0.001
	Intercept	-	2	<0.001
	Notification_5wk			
	Global model	0.0	16	0.620
	Spatial	1.0	12	0.380
	Environmental	30.7	6	<0.001
	Intercept	-	2	<0.001
	Notification+Confirmation			
	Global model	0.0	15	0.983
	Spatial	8.1	11	0.017
	Environmental	25.1	5	<0.001
	Intercept	-	2	<0.001
	Notification+Confirmation_5wk			
	Global model	0.0	16	0.719
	Spatial	2.0	12	0.262
	Environmental	7.3	6	0.019
	Intercept	-	2	<0.001
Survey6	Notification			
January 2021	Global model	0.0	16	0.904
	Spatial	4.5	12	0.096
	Environmental	18.3	5	<0.001
	Intercept	-	2	<0.001
	Notification_5wk			
	Global model	0.0	17	0.924
	Spatial	5.0	13	0.076
	Environmental	43.6	6	<0.001
	Intercept	-	2	<0.001
	Notification+Confirmation			
	Global model	0.0	16	0.957
	Spatial	6.2	12	0.043
	Environmental	20.6	5	<0.001
	Intercept	-	2	<0.001
	Notification+Confirmation_5wk			
	Global model	0.0	17	0.921

	Spatial	4.9	13	0.079
	Environmental	19.3	6	<0.001
	Intercept	-	2	<0.001
Survey7	Notification			
March 2021	Global model	0.0	18	0.938
	Spatial	5.4	14	0.062
	Environmental	27.8	5	<0.001
	Intercept	-	2	<0.001
	Notification_5wk			
	Global model	0.0	19	0.985
	Spatial	8.5	15	0.014
	Environmental	15.0	6	<0.001
	Intercept	-	2	<0.001
	Notification+Confirmation			
	Global model	0.0	18	0.551
	Spatial	0.5	14	0.433
	Environmental	7.1	5	0.016
	Intercept	-	2	<0.001
	Notification+Confirmation_5wk			
	Global model	0.0	18	0.8720
	Spatial	4.0	14	0.1202
	Environmental	9.5	5	0.0077
	Intercept		2	<0.001

Supplementary Table 2: A summary of entomological and epidemiological data gathering over the study period. Data is presented based in epidemiological weeks and denotes the corresponding activity performed on the corresponding week.

Epidemiological Week 2020	Beginning (dd.mm.yy)	Ending (dd.mm.yy)	Activity
1	29.12.19	04.01.20	-
...	...	...	-
9	23.02.20	29.02.20	-
10	01.03.20	07.03.20	Survey
11	08.03.20	14.03.20	Survey
12	15.03.20	21.03.20	1st week after survey
13	22.03.20	28.03.20	2nd week after survey
14	29.03.20	04.04.20	3rd week after survey
15	05.04.20	11.04.20	4th week after survey
16	12.04.20	18.04.20	-
17	19.04.20	25.04.20	-
18	26.04.20	02.05.20	-
19	03.05.20	09.05.20	Survey
20	10.05.20	16.05.20	Survey
21	17.05.20	23.05.20	1st week after survey
22	24.05.20	30.05.20	2nd week after survey
23	31.05.20	06.06.20	3rd week after survey
24	07.06.20	13.06.20	4th week after survey
25	14.06.20	20.06.20	-
